# Diversity, population genetics, and evolution of macrofungi associated with animals

**DOI:** 10.1080/21501203.2015.1043968

**Published:** 2015-05-18

**Authors:** Xiaozhao Tang, Fei Mi, Ying Zhang, Xiaoxia He, Yang Cao, Pengfei Wang, Chunli Liu, Dan Yang, Jianyong Dong, Keqing Zhang, Jianping Xu

**Affiliations:** a Laboratory for Conservation and Utilization of Bio-Resources, and Key Laboratory for Microbial Resources of the Ministry of Education, Yunnan University, Kunming650091, Yunnan, PR China; b Yunnan Institute for Tropical Crop Research, Jinghong, Yunnan, China; c Department of Biology, McMaster University, Hamilton, Ontario, CanadaL8S 4K1

**Keywords:** macrofungi, animals, interaction, population, coevolution

## Abstract

Macrofungi refers to all fungi that produce visible fruiting bodies. These fungi are evolutionarily and ecologically very divergent. Evolutionarily, they belong to two main phyla, Ascomycota and Basidiomycota, and many of them have relatives that cannot form visible fruiting bodies. Ecologically, macrofungi can be associated with dead organic matter, plants, and animals. Here we review our current understanding of population structure and biogeography of macrofungi associated with animals. Their interactions, functions, and patterns of coevolution are described and discussed. Our focus is on studies using molecular markers. Our analyses suggest that the types of fungi–animal associations play an important role in the structure of these animal-associated fungal populations.

## 1. Introduction

Macrofungi are an important component of Kingdom Fungi, and they play significant roles in natural ecosystems. Many of these fungi act both as key decomposers and as food sources for animals. Most macrofungi produce fleshy and colloidal fruiting bodies representing sexual reproductive structures; however, some visible structures, such as sclerotia, represent the asexual reproductive stage. Most macrofungi belong to Basidiomycota or Ascomycota while a few are Zygomycota. Their fruiting bodies may be located above- or below ground (Mueller et al. 2007). There is a large number of macrofungi in the world. Out of the approximately 100,000 described fungal species, an estimated 6000 can produce visible fruiting bodies and sclerotia (Ainsworth 2008). Macrofungi may live saprophytic, parasitic, and/or symbiotic lifestyles. Many of them, especially the symbiotic ones such as the majority of ectomycorrhizae, cannot reproduce independently. Instead, their host partners are needed to help them disperse and reproduce.

In this paper, we refer the partner organisms as those directly affecting the growth, development, dispersal, and reproduction of macrofungi. Our focus is macrofungi associated with animals. These associated animals may provide substrate, water, and ecological niches to macrofungi; or may help macrofungi reproduce by spreading spores and expanding niches; or may protect macrofungi from infection by other microbes. In return, these macrofungi provide food and/or protection for their associated animals. However, some macrofungi can also damage their partner animals through parasitism. Together macrofungi and their partner animals form a diversity of associations such as mutualism, parasitism, and cooperation. Because of their close relationships, these partner animals may have a significant impact on the life cycle, transmission, and reproduction of their associated macrofungi, including their genetic structure and evolution. In return, the population genetic structures of these fungi may also reflect the dispersal and reproductive patterns of their partner animals. For example, fungi associated with animals with long-distance dispersal abilities may show little or no geographic structure, while those associated with animals of limited dispersal abilities show significant geographic structure. Here we review the diversity of relationships between macrofungi and animals, with special emphasis on their interaction, genetic structures, and coevolution.

This review is divided into three parts based on the relationships between macrofungi and their partner animals. The first type of relationship is mutualism where macrofungi and animals live together and benefit from each other. The second type is parasitism where macrofungi uptake nutrients directly from animals while the animals receive no apparent benefit. The third type refers to a relatively loose association where the animals help macrofungi disperse spores that cannot be spread by wind, water, or other natural medium. For each type of relationships, we describe the representative organisms and emphasize the population genetics and evolution of these macrofungi. We mainly review studies using molecular markers. The main approaches, findings, and molecular markers are summarized in Tables 1 and 2. A general conclusion and perspective section is provided at the end of this review.

**Table 1.** Investigation strategies of genetic structure of macrofungi in mutualistic relationships with animals.

References	Species of macrofungi	Associated animal	Sampling site	Sampling time	Identification method(s)	Sample types	Population (sample) size	Makers/methods	Main result(s)
Nobre et al. 2011b	*Termitomyces* sp.	*Macrotermes bellicosus*	Pendjari National Park	–	Morphology of termites	Fungus combs or gut	24	ITS, EF1a, RPB2	Rare events of recombination
		*Macrotermes subhyalinus*	Pendjari National Park				9	ITS, EF1a, RPB2	Free recombination
De Fine Licht et al. 2006		*Macrotermes natalensis*	South Africa	2003.01 and 2004.01–03	Morphology of termites	Fungus combs	31	ITS, RPB2, RPB1, EF1a	Free recombination
Unpublished data		–	Yunnan China	2010.08 and 2013.08	Morphology and ITS	Fruiting bodies	18 (229)	RPB2, TEF, 12S, LACC	Free recombination
Vasiliauskas et al. 1998	*A. areolatum*	*Sirex juvencus*	Lithuania, Sweden, Denmark and Great Britain	–	Morphology	Wood or fruit bodies or glands of woodwasps	53	M13 mini satellite and somatic compatibility	Dispersing clonally
	*A. chailletii*	*Urocerus gigas*					57		Spreading by basidiospores
Margrete Thomsen and Koch 1999	*A. areolatum*	*Sirex juvencus*	Denmark (more than 100 km)	1992–1995	Morphology	Basidiocarps, wood isolations, woodwasps	31	Somatic compatibility	High degree of somatic compatibility between isolates
	*A. chailletii*	*Urocerus gigas*					69		Rare Somatic compatibility between isolates
Liang et al. 2008	*O. sinensis*	Ghost moths	Qinghai, Tibet, Yunnan, and Sichuan province of China	–	Morphology	Fruiting bodies	18(180)	ISSR (9)	Little gene exchange, latitudinal genetic differentiation
Zhang et al. 2009	*O. sinensis*	Ghost moths	Tibet, Qinghai, Sichuan and Yunnan	During 2005 and 2008	Morphology	Fruiting bodies	11(56)	ITS, *MAT*1-2-1	Greater genetic diversification among southern isolates
			Province in China						
Quan et al. 2014	*O. sinensis*	Ghost moths	Qinghai, Tibet, Sichuan, Gansu, Yunnan provinces	–	Morphology	Complex of *O. sinensis* stromata and host cadavers	33	ITS, β-tubulin	Similar phylogenetic relationships and genetic structure
	Hepialidae caterpillars	–						COI, COII, Cytb	
Wang et al. 2008	*C. militaris*	–	Guangdong, Hebei, Liaoning, Jilin in China and Seoul in Korea	–	Morphology	Fruiting bodies and fresh mycelia	(13)	ITS, RAPD	Extremely small genetic variation
Wen et al. 2012	*C. militaris*	–	Sichuan province of China	–	Morphology	Fresh mediums	24	RAPD, *MAT*	Genetic variation of different monoconidial isolates
Rubini et al. 2005	*T. magnatum*	–	Italy, Croatia and Slovenia	–	Morphology	Ascomata	26 (316)	SSR	Positive correlation between genetic and geographical Distances
Bertault et al. 1998	*T. melanosporum*	–	France and Italy	–	Morphology	Ascocarps	12 (208)	RAPD	Low level of polymorphism
Murat et al. 2004	*T. melanosporum*	–	France, northern Italy and north-eastern Spain	1998.12–2003.02	Morphology	Fruit bodies	17 (188)	RAPD, ITS, SCAR	Significant genetic differentiation between regional populations
Riccioni et al. 2008	*T. melanosporum*	–	Italy, France, Spain	2000–2006	Morphology	Ascocarps, hyphal fragments	13 (210)	SSR, ITS, AFLP	Geographic differentiation among populations
García-Cunchillos et al. 2014	*T. melanosporum*	–	Iberian Peninsula	–	Morphology	Ascocarps	23 (190)	SSR	High levels of genetic diversity
Wang et al. 2006a	*T. indicum* complex	–	Huidong, Kunming Gongshan, Miyi, Panzhihua, Huili	2003, 2004 and 2005	Morphology and ITS	Ascocarps	6 (30)	ITS	Genetic differentiation among regional populations
							5 (26)	β-tubulin	
Murat et al. 2013	*T. melanosporum*	–	Montemartano and Rollainville	2010–2011	Morphology and ITS	Soil, ascocarps and ectomycorrhizal roots	10	MAT, SSR	spatial genetic structure

Note: RAPD, random amplified polymorphic DNA.

**Table 2.** Common molecular markers and for representative macrofungi.

Species	Targets	Primer name	Primer sequence	References
*Termitomyces* sp.	mtSSU-rDNA	ssufw105	TCGCGTTAGCATCGTTACTAGT	Aanen et al. 2002
		ssurev475	GCCAGAGACGCGAACGTTAGTCG	
	EF1a	EF595F	CGTGACTTCATCAAGAACATG	Nobre et al. 2011b
		EF1160R	CCGATCTTGTAGACGTCCTG	
	RPB2	RPB2-tF	GCG(GA)CGGAAAGACGACATCAG	
		RPB2-tR	TTGTGATCAGGGAATGGGAT	
	RPB2	bRPB2-6F	TGGGGYATGGTNTGYCCYGC	De Fine Licht et al. 2006
		bRPB2-7	CCCATRGCYTGYTTMCCCATDGC	
	RPB1	RPB1-AF	GARTGYCCDGGDCAYTTYGG	
		Frpb1-CR	CCNGCDATNTCRTTRTCCATRTA	
	EF1α	EF634F*	AGGCTGACTGCGCTATCCTTAT	
		EF1127R*	GGTTCGATGGCATCGATGGCAT	
*A. areolatum*	nuc-IGS-rDNA	P-1	TTGCAGACGACTTGAATGG	Nielsen et al. 2009
		5S-2B	CACCGCATCCCGTCTGATCTGCG	
	RPB2	bRPB2-6F	TGGGGYATGGTNTGYCCYGC	Bergeron et al. 2011
		bRPB2-7.1R	CCCATRGCYTGYTTMCCCATDGC	
	TEF1	tef1f	TCMAHGARATYATYAAGGAGAC	
		tef1rc	DGGGTCGTTYTTSGAGTCA	
	LAC-like	laccasef	CACTGGCACGGNTTCTTCCA	
		laccaserc	GTGACTATGATACCAGAANGT	
	MAP	MIPAro1F*	GTCCTTTCACTCTTCGGTAC	Van Der Nest et al. 2008
		MIPAro1R*	CAAATAACTGGCGCCATACC	
	PAB1	RAB1-470F*	TCTTGGGCTGACTTTTCC	
		RAB1-1800R*	GGCAGGTAGATCGAGGTTGA	
	*mat*-B	br1-F	TGGCATMTNCARGCNTGGAAYTC	
		br1-1R	GCGAGNRNCATNAGNCGNAKGTA	
Septobasidiaceae fungi	TEF-1	SEF1a1fI	CTYGGIAAGGGITCNTTCAAG	Henk and Vilgalys 2007
		SEF1a1r2	CATICCGGCCTTGATNGTNCC	
*O. sinensis*	rDNA	OsT-F*	GTCAAGAAGCAAGCAAAGGAATC	Zhong et al. 2014
		OsT-R*	TCAACTGGAGGGTGTGGTGG	
	*MAT*1-2-1	Mat1-2F*	TGGAATGCGACTGACTACGA	Zhang et al. 2011**
		Mat1-2R*	CCAGGAGAGCTTGCTTGACT	
	*MAT1-1-1*	MATF2	AAACGCCCTCTCAAYGCNTTYATG	Bushley et al. 2013**
		MATR3	CCACTTGCTTCTGAANGGRTCYTTRTTCCA	
	DNA lyase	DNAF3	TTGATGGGATCCGAYCAYTGYCC	
		DNAR2	GTGACCAGGCTGATRCANGGYTC	
	MAT1-1-3	M3F1	CAGCAGCCGGTGAAGGTNTWYCAYGA	
		M3R1	CTTCGTTGTGTACTTGTANYCNGGRTA	
	β-Tubulin	T1	AACATGCGTGAGATTGTAAGT	Quan et al. 2014
		T22	TCTGGATGTTGTTGGGAATCC	
	SSU rRNA	NS1	GTAGTCATATGCTTGTCTC	Zhong et al. 2010**
		NS4	CTTCCGTCAATTCCTTTAAG	
*C. militaris*	*MAT*1-1-1	MAT1-F1	CGRGCWAARCGRCCATTKAAYGC	Yokoyama et al. 2006**
		MAT1- R1	TTKCCCATCTCRTCRCGGAYRAARGA	
	MAT1- 2-1	MAT2 -F1	GCRTATA TTCT RT ACCGC AG	
		MAT2-R1	CGAGGTT GATAYTGAT AYT G	
*T. indicum* complex	β-Tubulin	Bt2a	GGTAACCAAATCGGTGCTGCTTTC	Wang et al. 2006a
		Bt2b	ACCCTCAGTGTAGTGACCCTTGGC	
*T. melanosporum*	MAT1-1-1	P19*	CAATCTCACTCGTGATGTCTGGGTC	Rubini et al. 2011b**
		P20*	TCTCGGGCTGGAGGTGCGGGTCGAGT	
	MAT1-2-1	P1*	CAGGTCCGTCATCTCCTTCCAGCAG	
		P2*	CCACATGCGACCGAGAATCTTGGCTA	
*Tuber* truffles	EF1α	Tuber_f*	AGCGTGAGCGTGGTATCAC	Bonito et al. 2013**
		Tuber_r*	GAGACGTTCTTG ACGTTGAAG	
	RPB2	Tuber_f*	YAAYCTGACYTTRGCYGTYAA	
		Tuber_r*	CRGTTTCCTGYTCAATCTCA	
	PKC gene	pkc1f	CCCAAAGGTGGTCACGAAGTGTA	Wang et al. 2006b**
		pkc1r	TGATGAACTCCTTCTTCAGAACC	
	β-Tubulin	Bt1a	TTCCCCCGTCTCCACTTCTTCATG	
		Bt1b	GACGAGATCGTTCATGTTGAACTC	

Note: *The special primers; **there are other primers for the target in the reference. The references of black body present available universal or special primers of ITS.

## 2. Macrofungi in mutualistic relationships with animals

German mycology founder de Bary first used the word “symbiosis” to refer to different organisms living together closely (Goff 1982). Forms of symbiosis include parasitism, mutualism, commensalism, amensalism, and proto-cooperation. In this section, we review representative examples of macrofungi in mutualistic relationships with animals.

### 2.1. *Termitomyces* and fungus-growing termite (Macrotermitinae)

The relationship between the Basidiomycete genus *Termitomyces* (Lyophyllaceae) and the fungus-growing termites of Macrotermitinae is among the best-known examples of obligate symbiosis between fungi and animals. Traditionally, the macrofungi observed in termite nests were classified to genera *Lentinus, Entoloma, Pluteus, Armillaria,* and so on until Heim (1942) proposed the genus *Termitomyces* to accommodate them. Researchers have ascertained that these fungi and termites can help each other adapt to variable environmental conditions through their mutualistic interactions (Rouland-Lefèvre et al. 2006). In these interactions, the termites provide a suitable microclimate and substrate to the fungi, and selectively inhibit other fungal competitors and microbial infections by secreting specific substances. In return, the fungi provide nitrogen-enriched nutrients to the termites, as well as enzymes that help the termites to obtain additional food (Korb and Aanen 2003). Incidentally, products of this mutualistic relationship provide benefits to humans, too, by supplying us gourmet and nutritive mushrooms for our dining table. Ecologically, these macrofungi help decompose cellulose, semi-cellulose, and chitin, facilitating nutrient cycling in soil and enriching soil fertility.

Recent researches suggested that the termite–macrofungi association likely originated in the African rain forest and had at least four independent “out-of-Africa” migrations into Asia, and one independent migration into Madagascar (Aanen and Eggleton 2005; Nobre et al. 2011a). However, the termite–macrofungi association is now widespread and has been reported in southern Africa, South Asia, Southeast Asia, and the South Pacific islands (Wang et al. 2012). The spread of the termite–fungal associations across geographic regions was likely mediated by the termites. As the termites disperse, they bring their associated fungi (spores, hyphal fragments, and spherules) with them. This type of dispersal maintains the same genotypic associations (barring mutations) of the original nest. Over time, this vertical transmission would result in a co-phylogenetic pattern between the termites and their associated fungi. Indeed, such a pattern has been observed in several studies. For example, the single colonization of *Termitomyces* fungi in Madagascar was likely established through this mechanism (Nobre 2010a; Nobre et al. 2010b). In addition, Nobre et al. (2011b) found that the genetic structure of symbiotic fungi of *Macrotermes bellicosus* is consistent with clonal reproduction with limited evidence for recombination (Table1). These observations indicated that the fungus-growing termites play a significant role in the distribution and genetic structure of their associated *Termitomyces* fungi.

Aside from vertical transmission and local reproduction in natural populations of the *Termitomyces* fungi, evidence for horizontal transfer of fungi between termite nests has also been found for these fungi (De Fine Licht et al. 2006). Horizontal transmission occurs when worker termites forage fungal basidiospores and establish new nests. The horizontal pattern can facilitate transmission of fungal haplotypes and species among hosts (Osiemo et al. 2010), creating opportunities for mating and recombination. Indeed, evidence for recombination among populations was found in investigations of *Macrotermes natalensis* and its *Termitomyces* fungi (De Fine Licht et al. 2006) and our studies in Yunnan, China (unpublished data) (Table1).

Through horizontal transmission, a given fungal species can establish symbiotic relationship with several termite species within a genus (Aanen et al. 2002). Evidence for the dominant role of horizontal transmission of *Termitomyces* genotypes has been found in sequence analyses of *Termitomyces*. For example, Osiemo et al. (2010) found that there were 41 *Termitomyces* lineages in Africa and that only 40% of the internal transcribed spacer (ITS) sequence variations were due to the separations based on host genera. The remaining 60% of sequence variations were found within individual host genera. The large contribution of the host suggests long-term stability and potential specificity of the *Termitomyces*–host termite relationships. Indeed, vertical transmission of *Termitomyces* fungi along the host lines can be easily maintained stability and accomplished by simply carrying the fungal culture from the original nests. However, because each new termite nest of horizontal transmission contains abundant fungal spores, selection must be operating within individual nest where favorable strains/genotypes could be selected by termites (Frank 1996).

The selectable traits in fungi by termites are likely related to flavor and nutrients within the mycelia. The termites consume fungal asexual fruiting bodies (spherules) as food. Aside from being food themselves for termites, these fungi secrete lignocellulolytic enzymes, which mix with enzymes of the termites and possibly bacteria from the termite gut or nest to degrade foraged plant material efficiently. Therefore, a fungal strain could be selected as monoculture if it provided superior nutrients for its host to grow more competitively (Nobre and Aanen 2012). In the fructiferous nests, termites only select superior fungal strains from two spores with different mating types. The fact that host termites contribute significantly to fungal genetic variation suggested that selection within termite nests rather than between nests has likely played the dominant role in nature. Fruiting and sexual recombination within nests can produce abundant recombination genotypes from which selection could occur.

It is estimated that the termites started cultivating symbiotic fungi for food about 30 mya ago (Nobre et al. 2011b). Duringer et al. (2006) reported the first fossil fungal combs extracted from a 7-million-year-old continental sandstone, the oldest symbiotic termite fungiculture known at that time. More ancient fossil evidences were subsequently reported (Duringer et al. 2007), further demonstrating the long-term association between symbiotic fungi and termites. By comparing the phylogenetic tree of 15 African *Termitomyces* species with the taxonomic tree of Macrotermitinae, Rouland-Lefevre et al. (2002) found that a *Termitomyces* species can associate with different termite species within the same genus. Furthermore, by analyzing the relationships between several termite species from Asia and their associated *Termitomyces* fungi, Taprab et al. (2002) discovered that the symbiotic fungal species and their distribution paralleled those of the termites. Similarly, Aanen et al. (2002) investigated 32 termite species in 9 genera from Asia and Africa and their associated *Termitomyces* fungi. The results showed that the symbiosis has a single African origin, with no evidence for secondary domestication of other fungi or the reversal of mutualistic fungi to a free-living state. The two phylogenetic trees, one for termites and the other one for fungi, both showed five main clades with an one-to-one correspondence (Aanen et al. 2002). Together, these studies suggest that major splits have occurred simultaneously in the termites and fungi in their long co-evolutionary process.

Aside from the co-evolutionary association between the *Termitomyces* and termites, termite nests or the gut of termites often contain non-*Termitomyces* fungal and bacterial communities (Moriya et al. 2005; Mathew et al. 2012). The bacterial community has shown capable of helping to degrade lignin and inhibit the non*-Termitomyces* fungal competitors. For example, *Bacillus* spp., the dominant bacterial species in termite nests, can help degrade lignin and inhibit the filamentous contaminant fungus *Trichoderma harzianum*, a competitor of *Termitomyces* (Mathew et al. 2012). Similarly, fungi in the genus *Xylaria* are also commonly found in termite nests (Visser et al. 2011). The *Xylaria* fungi do not cause any obvious damage to the nest or *Termitomyces* fungi but can take over the nest when the termite colony dies. The contributions of the bacteria and non*-Termitomyces* fungi to the evolution of Termites–*Termitomyces* relationships remain to be determined.

### 2.2. *Amylostereum* and woodwasps

The genus *Amylostereum* (Basidiomycota) was proposed in 1958 by Boidin to accommodate species with smooth amyloid basidiospores, hyaline-encrusted cystidia, and resupinate to effuso-reflexed fruiting bodies in the genus *Stereum*. Since then, several revisions have been made and the current classification of the genus includes four species: *Amylostereum chailletii, Amylostereum areolatum, Amylostereum laevigatum,* and *Amylostereumferreum* (Slippers et al. 2003). The first three species can form a fascinating mutualistic association with various species of siricid woodwasps (Gaut 1970; Tabata and Abe 1999). In these mutualistic associations, the female woodwasps have two special features: (i) the intersegmental sacs, which can carry fungal spores and deposit them when their eggs are laid in host trees; and (ii) the phytotoxic substances that the woodwasps secrete to help create favorable conditions for the fungi (Thomsen and Harding 2011). In return, the fungi provide a suitable environment, nutrients, and enzymes that are necessary for the survival and development of the insect larvae (Slippers et al. 2003). In general, the woodwasps spend most of their life as burrowing larvae in host trees while adult wasps live only for a few weeks to mate and lay eggs in other trees every one to three years (Benson 1943; Morgan 1968). This type of insect–fungal complex does not usually result in obvious economic and ecological damage. However, host trees may be harmed by the burrowing activity of the siricid larvae and by the wood-degrading activities of the *Amylostereum* fungi. Indeed, the complex between *Sirex noctilio* and *A. areolatum* has caused extensive mortality of commercial wood species in the southern hemisphere (Berryman 1988; Chou 1991). Therefore, much of research activities on this group of fungi have been initiated for the protection of the forestry industry in the southern hemisphere.

Studies have shown that this insect–fungus complex is native to the northern hemisphere, where a natural balance exists among the insect–fungus complex, its natural parasites and host trees. However, in the last century, the organism complex was introduced into various countries in the southern hemisphere and created a big problem for the forestry industry (Slippers et al. 2003). The spread was likely carried out by the woodwasps, along with the fungal oidia over large areas. Using traditional and molecular methods, researchers now have proven that isolates of *A. areolatum* from different regions are genetically related. It was found that the isolates of *A. areolatum* from various countries in the southern hemisphere have complete or partial vegetative compatibility (Slippers et al. 2003). Vegetative compatibility is determined by multiple genes collectively at the heterokaryon incompatibility (*het*) loci (Van Der Nest et al. 2008) with each locus having at least two different alleles. Isolates with different allele(s) at any of the loci would result in vegetative incompatibility. Similarly, based on nucleotide sequence data at the intergenic spacer region of the nuclear ribosomal DNA, Nielsen et al. (2009) found that similar genotypes of *A. areolatum* exist in both Europe and Northern America. Furthermore, Bergeron et al. (2011) found that two multilocus genotypes of *A. areolatum* in the southern hemisphere were the same as those from the northern hemisphere at the sequenced mitochondrial and nuclear genes.

Comparisons between the phylogenies of the woodwasps and their associated *Amylostereum* fungi indicated little species-specific relationships between the two interacting partners (Hajek et al. 2013). For example, *A. areolatum* can be carried by various wasp species, while *Sirex nigricornis* and *Sirex nitidus* can be found associated with either *A. chailletii* or *A. areolatum* (Gaut 1970; Hajek et al. 2013). Therefore, the *Amylostereum* fungi have shown themselves capable of finding new insect partners in various conditions.

The flexible relationships between the two interacting partners suggest that genetic exchange among strains within individual fungal species is possible. Genetic exchange and recombination require mating between genetically different strains. *Amylostereum* species are heterothallic (Boidin 1958) and they can be spread via sexual basidiospores (by either wind or woodwasps) and asexual oidia (by woodwasps). The relative roles of two types of spores spread by wasps vary among the fungal species. Vasiliauskas et al. (Vasiliauskas et al. 1998; Vasiliauskas and Stenlid 1999), Margrete Thomsen and Koch (1999) (Table 1), and Slippers et al. (2005) investigated the genetic structure of *A. areolatum* and *A. chailletii*. They found that the isolates of both *A. areolatum* and *A. chailletii* can be spread clonally by woodwasps. However, *A. areolatum* exhibits a higher degree of clonality in both its native habitats and the newly invaded southern hemisphere. For example, the same genotype and the same vegetative compatibility groups (VCGs) of *A. areolatum* were found broadly distributed worldwide. In contrast, the population structure of *A. chailletii* suggested more evidence for sexual recombination, consistent with a more important role of basidiospores in its dispersal than that in *A. areolatum*. Furthermore, only a small fraction of *A. chailletii* isolates from diverse areas shared the same genotypes and the distributions of such genotype sharing decreased with increasing geographic distance.

The understanding of the interactions between fungi and wasps has been used to develop biocontrol measures. For example, *Deladenus siricidicola* parasitizes *S. noctilio* woodwasps and feeds on *A. areolatum*, thus playing an important role in maintaining the population balance of various organisms in nature. Recently, *D. siricidicola* was used as a biological control agent of woodwasps with varying degrees of success (Erin Morris et al. 2014). Similarly, the *Amylostereum* fungi have been found capable of influencing the feeding and reproductive ability of certain nematodes. The nematodes have differing abilities to persist on different *Amylostereum* species and isolates. For example, *D. siricidicola* nematodes were able to reproduce when feeding on both native and invasive strains of *Amylostereum* fungi (Erin Morris et al. 2014). However, strains of *D. siricidicola* grew differently when feeding on different isolates of *A. areolatum* (Morris et al. 2012). In addition, Hurley et al. (2012) discovered that the competitive interaction between *A. areolatum* and sapstain fungi negatively influenced the success of *D. siricidicola*. The *Amylostereum* fungi, woodwasps, and parasitic nematodes compose a complex community that needs further study.

### 2.3. *Septobasidium* and scale insects (Diaspididae)

*Septobasidium* (Patouillard) is the largest genus in Septobasidiaceae, which is the only family under Septobasidiales. Members of the *Septobasidium* genus have close associations with scale insects. Currently, Septobasidiaceae contains 5 genera with approximately 175 species, in which the majority are placed in the genus *Septobasidium*. These fungi either live in a putatively altruistic mutualism with scale insects (Coccoidea) or parasitize insects. Other closely related fungi include those in the genus *Uredinella*, considered to be an intermediate group between *Septobasidium* and the rust fungi (Couch 1938, 1941). While molecular phylogenetic analyses suggested a single origin of Septobasidiales (Septobasidiaceae), there has been little evidence to support *Septobasidium* as a monophyletic group within Septobasidiaceae (Henk and Vilgalys 2007). We here only review the association between *Septobasidium* and scale insects.

Although the genus *Septobasidium* was established by French mycologist Narcisse Théophile Patouillard in 1892, the interaction between these fungi and scale insects retained unknown until Couch’s studies were published (Couch 1938). Generally, these fungi infect scale insects to obtain necessary nutrients and grow as mats of hyphae out of the insects to cover infected and uninfected insects on branches and leaves. The infected insects lose the abilities to move and reproduce but they can still get nutrients from the tree leaves. Through this interaction, the *Septobasidium* fungi sterilize the insects that they parasitize to get nutrients while their mats of hyphae can protect certain uninfected insects from parasitoid wasps and adverse environmental conditions. However, their interaction causes the common plant disease called patch cankers, where the fungi grow around branches of trees like a white or brown sparadrap. The protection afforded by the fungi to uninfected scale insects makes it easier for the scale insects to parasitize and harm plants, especially certain fruit crops, so studies in this field are important for agriculture and forestry.

Couch (1938) also found that the scale insects were essential for reproduction and dispersal of *Septobasidium*. He found that the *Septobasidium* fungi could not produce basidiospores on laboratory substrate without scale insects. In addition, the fungi can grow other structures as responses to scale insects in nature (Cheng 2013). The scale insects are also the main vectors to disperse basidiospores of *Septobasidium* with spores of *Septobasidium* adhering to insects to move to other places. Their similar geographic distributions are consistent with the observed dispersals of the *Septobasidium* fungi and the scale insects (Henk and Vilgalys 2007). Even though the fungal genus and the scale insects are broadly distributed, individual species within either the fungi or the scale insects show obvious geographic patterns with the tropic and temperate zones exhibiting significant differences (Cheng 2013). Recent researches also identified significant undescribed diversity of *Septobasidium* fungi. Indeed, major revision with regard to the taxonomy and systematics of the *Septobasidium* fungi are needed (Gómez and Kisimova-Horovitz 2001; Gómez-Pignataro and Henk 2004; Chen and Guo 2012).

## 3. Macrofungi parasitizing animals

Parasitism is an important pattern of fungal–animal interactions. There are many parasitic fungi distributed widely in every subdivision of the kingdom fungi. However, macrofungi parasitizing animals are rare and not well known. The cordyceps fungi are a group of parasitical macrofungi that mainly parasitize insects, other arthropods, and other fungi (Zhang et al. 2012). Up to now, over 400 species of cordyceps fungi have been reported and most of them have been valuable traditional Chinese medicine and food for centuries (Chen et al. 2013). All of the cordyceps fungi were originally assigned to the genus *Cordyceps*, family Clavicipitaceae (Kirk et al. 2001). However, it has been found recently neither Clavicipitaceae nor genus *Cordyceps* were monophyletic. The revised taxonomy has the cordyceps fungi assigned to three families (Clavicipitaceae, Cordycipitaceae, and Ophiocordycipitaceae) and five genera (*Cordyceps, Elaphocordyceps, Metacordyceps, Ophiocordyceps,* and *Tyrannicordyceps*) (Sung et al. 2007). The best-known *Ophiocordyceps sinensis* (Chinese caterpillar fungus) and *Cordyceps militaris* are assigned to genera *Ophiocordyceps* and *Cordyceps* respectively now.

### 3.1. Ophiocordyceps sinensis

The Chinese caterpillar fungus, which had been assigned to *Cordyceps* until Sung et al. (2007) assigned it to the genus *Ophiocordyceps*, is the most famous and best-known cordyceps fungi. It mainly parasitizes larvae of ghost moths, which commonly dig and live in underground tunnels (Zhang et al. 2012). Generally, the anamorph of *O. sinensis, Hirsutella sinensis* (Li et al. 2006), typically live around plant roots that are also the food source for the ghost moths, thus creating opportunities for the fungus to infect the ghost moths (Zhong et al. 2014). Most infections occur in late autumn and the fungi can infect host larvae in three ways: (i) the fungal hyphae penetrate the spiracles of larvae; (ii) the ascospores adhere to the cuticles of larvae; or (iii) the host larvae ingest the fungal ascospores (Zhang et al. 2012). Subsequently, the fungi develop inside the larva and kill it. However, the infected larvae can still move beneath the soil surface before dying. In the summer after 2–3 years, a fungal stroma (fruit body) emerges from the head of the dead host larva (Zhang et al. 2012). The fungi–larva complex is the famous Chinese traditional medicine called “Dong Chong Xia Cao,” which means grass in the summer worm in winter. This Chinese cordyceps is well known for its immunomodulatory, antitumor and antiaging effects. It is also among the most expensive traditional Chinese medicine, with high-quality wild cordyceps worth more than its weight in gold. However, due to our inability to artificially cultivate the Chinese cordyceps, the high price has resulted in overexploitation and habitat degradation, leading to declining natural populations and harvest. Over the last few years, there have been significant efforts by the scientific community to understand the diversity, ecology, and evolution of *O. sinensis* in an effort to develop better for conservation, utilization, and cultivation strategies.

The Chinese cordyceps distributed only at high altitude of 3000–5100 m above sea level on the Tibetan Plateau. The geographic areas include Tibet, Qinghai, and the western parts of Gansu, Sichuan, and Yunnan provinces in China and the southern regions of the Himalayas in Nepal and northern India (Li et al. 2011). Similarly, its hosts, the ghost moths, are also endemic in these regions. The genetic variation with *O. sinensis* shows strong geographical patterns. First, *O. sinensis* presents a latitudinal genetic differentiation from south to north in its geographical distribution range and can be divided into northern, middle, and southern populations. Second, the genetic diversity and diversification among isolates in the south is greater than those in the north (Liang et al. 2008; Hao et al. 2009; Zhang et al. 2009) (Table 1), which have led to the suggestion that the Nyingchi District of southern Tibetan Plateau is the center of origin of *O. sinensis*. Third, *O. sinensis* showed substantial intraspecific genetic diversity and evidence of speciation (Zhang et al. 1999, 2009; Liang et al. 2008). Fourth, though evidence of recent gene flow has been found between geographic populations, the mountains ranges or river system have shown influences on population structures among geographic populations. Last but not the least, the distribution of host ghost moths has also been found to have a big influence on the genetic relationships between *O. sinensis* populations (Zhou et al. 2007; Liang et al. 2008; Quan et al. 2014) (Table 1). It is estimated that *O. sinensis* has over 50 host species. These ghost moth species are all endemic in the Qinghai-Tibet Plateau and most species have narrow geographical distribution (Yang et al. 1996; Wang and Yao 2011). While certain genotypes of *O. sinensis* were found to have broad host ranges, many were found to be only associated with a specific host genotype/species. The importance of hosts in *O. sinensis* is also shown in their reproductive life cycles of both *O. sinensis* and the ghost moths. Although the asexual reproduction and the infection mechanism remain unknown, the sexual reproduction of *O. sinensis* only occurs after infecting hosts (Stone 2008). Indeed, evidence of co-speciation has been found between the ghost moths and *O. sinensis* (Zhang et al. 2014). These results suggest that conservation efforts of *O. sinensis* should also take into account the geographic distribution of host insects.

In addition to *O. sinensis* and its host insects, other biotic and abiotic factors have likely played very important roles in the evolution of the ghost moths–*O. sinensis* parasitoidism associations. The biotic factors include the plants and a microbial community of fungi, bacteria, and actinomycetes in the microenvironment of natural *O. sinensis*. For example, more than 200 fungal operational taxonomic units were detected from natural *O. sinensis* and some of them can produce chemical and pharmaceutical components similar to those of *O. sinensis* (Zhang et al. 2012). These fungi may affect life cycle of *O. sinensis* and have potential application in the artificial cultivation of Chinese cordyceps. In addition, it was investigated that bacteria, fungi, and actinomycetes occur frequently in the intestines of host larvae; and the frequency of occurrence and abundance of species are different among the intestines of wild larvae, laboratory-reared larvae, and mummified larvae (Liu et al. 2008; Yu et al. 2008; Zhang 2009). These microorganisms may influence the growth and development of larvae or as pathogens to interfere with the large-scale rearing of host larvae for the commercial cultivation of Chinese cordyceps (Zeng and Yin 2003). The effect of these microorganisms on growth and development of *O. sinensis* and host larvae requires further investigation.

### 3.2. Cordyceps militaris

*C. militaris* is the type species of the genus *Cordyceps*. It has been researched for a long time, and its metabolomics (Choi et al. 2010), genome (Zheng et al. 2011), transcriptome and proteome (Yin et al. 2012) have been reported. Its total genome size is 32.2 Mb and 16% protein coding genes showed differential expression between the in vitro and in vivo cultures and are likely related to interactions with its host insects. *C. militaris* have broad host ranges and can parasitize about 70 insect species in 17 families, including 12 families within Lepidoptera, 3 within Coleopteran, and 1 family each within Hymenopteran and Dipteran (Shrestha et al. 2012). Unlike *O. sinensis* that only parasitize larva, the *C. militaris* can parasitize larva, pupa, and adult of host insects, with most infections occurring on the pupa (Chen 1986). In addition, multiple stromata of *C. militaris* often emerge from different positions of a single pupa. As a result, the insect–*C. militaris* association has been called “pupa grass” in China. Compared to *O. sinensis, C. militaris* grows faster and can be artificially cultivated. Chemical analyses have shown that *C. militaris* have similar medicinal properties as *O. sinensis*. Therefore, the large-scale production of stroma of *C. militaris* has been used as a substitute for *O. sinensis*. However, the mechanism of how *C. militaris* infects pupae and grows well under artificial conditions remains poorly understood (Shu et al. 2013).

*C. militaris* is distributed at altitudes lower than *O. sinensis*, from 0 to >2000 m above sea level. It has been reported in many parts of the world, from North America, South America, Europe, and Asia (Shrestha and Sung 2005; Ma et al. 2007). This wide distribution was likely due to its diverse host species and their wide geographic distribution. Several population genetic analyses of *C. militaris* have been reported, and these studies have identified limited genetic differentiation among geographic populations of this species. For example, genetic analyses using RAPD marker found little correlation between the genetic variation and geographic regions of *C. militaris* among 11 sites in South Korea (Sung et al. 1999); similarly, Wang et al. (2008) also found that less than 1% of the genetic variation was attributable to geographic separations for *C. militaris* populations from Britain, China, Japan, Korea, and Norway (Table 1). At smaller geographic scales, Wen et al. (2012) found that different monoconidial isolates showed genetic variation, consistent with genetic recombination and heterothallism of *C. militaris* (Table 1). The mating system in *C. militaris* is controlled by one locus with two alleles, *MAT*1-1-1 and *MAT*1-2-1 (Shrestha et al. 2004; Yokoyama et al. 2006; Zheng et al. 2011). While natural fruiting bodies are formed from insects infected with heterokaryotic mycelia containing both mating types, fruiting bodies can also form on cereal substrate (Shrestha et al. 2012). Therefore, the host insects seem unnecessary for the sexual life cycle of *C. militaris*. However, Xiong et al. (2010) found that the transcriptional profiles of fruit bodies produced on rice medium and silkworm pupae are different. Zhang et al. (2013) also suggested that the pharmaceutically active ingredients are different between fruiting bodies produced in artificial medium and artificial Lepidoptera pupae or wild.

## 4. Macrofungi that require animals for dispersal

In this section, we review representative population genetic studies of fungi that are loosely associated with animals, which contribute to the dispersal of fungal spores or mycelia. In this type of association, the animals and macrofungi live independently most of the time. However, at certain stages, they interact with each other. For example, the fungal fruiting bodies may be a source of food for the animal and the animals help macrofungi to spread spores as a by-product of the feeding. Without the help of the animals, these macrofungi may have limited opportunities to spread. Next we divide these macrofungi into three groups to describe and discuss them: (i) underground or hypogeous fungi, (ii) stinkhorn fungi, and (iii) coprophilous fungi.

### 4.1. Hypogeous macrofungi

The hypogeous macrofungi can produce large fruiting bodies, which develop under the surface of soil or being covered by a thick layer of humus or leaf litter (Hawker 1954). Most hypogeous fungi can form ectomycorrhiza with plants from which they obtain carbohydrates. In return, these macrofungi help plants by providing mineral nutrient and water and protecting them from root pathogens. These macrofungi are also important foods of small mammals. Some of the hypogeous macrofungi are also a significant source of income for humans. On their own, the hypogeous macrofungi have very limited ability to disperse, for example, by hyphal extension and shedding spores, which may be transported to a nearby location by soil-inhabiting invertebrates and small mammals (Hawker 1954). The long-distance dispersal of these fungi is accomplished when the mycophagous mammals dug up and consume underground sporocarps and then defecate the spores at other places (Claridge et al. 2000). Thus, the mycophagous mammals can have a significant influence on the reproduction, transmission, and genetic structure of hypogeous macrofungi.

Hypogeous macrofungi are broadly distributed into three fungal phyla, the Basidiomycota, the Ascomycota, and the Zygomycota. The economically important species are found mostly in Hymenogastrales (Basidiomycetes) and Tuberales (Ascomycetes). The black truffle belongs to Tuberales and is often referred to as the “black jewel” of European dining tables. As a result, the ascocarps of the genus *Tuber* (true truffle) have been studied extensively for their genetic structure and fungi–animal interactions.

*Tuber* is the monophyletic truffle genus in Tuberaceae that includes truffle and non-truffle species. The genus evolved from an epigeous ancestor and dispersed with host plants’ migration (Bonito et al. 2013). Currently, there are over 200 species in this genus (Murat et al. 2013). Similar to other hypogeous macrofungi, *Tuber* truffles require and recruit mycetophagous mammals to disperse their spores. Generally, mycetophagous animals are attracted by truffle volatiles, which then consume sporocarps and disseminate spores in their fecal pellets (Splivallo et al. 2011; Danks 2012). In the case of truffles, the dispersing distance is determined by two factors: (i) the gut-retention time of spores in mycetophagous mammals, which generally might be more than 20 h; and (ii) the travel distance of the mammals within that time span, which may cover dozens of hectares (Danks 2012; Vernes and Jarman 2014). The mycetophagous mammals help *Tuber* species to disperse and by association increasing the health and productivity of host plants.

*Tuber melanosporum* and *Tuber magnatum* are two highly prized truffles in Europe. They are the favorites of gastronomers and businessmen, and can be cultivated semiartificially by inoculation of young trees and plantations. The two species have similar genetic structure in their geographical distributing range. In the most productive areas of Italy and Istria peninsula, *T. magnatum* showed a significant positive correlation between genetic and geographical distances and that the southernmost and northwesternmost populations were significantly differentiated from other populations (Rubini et al. 2005) (Table 1). *T. melanosporum* had also low-level genetic diversity across its geographical range of France and Italy (Bertault et al. 1998) (Table 1) but presented significant geographic differentiation among populations (Murat et al. 2004; Riccioni et al. 2008) (Table 1). The southernmost populations of *T. melanosporum* also showed higher allelic richness than the northern populations (Riccioni et al. 2008). These similar population genetic characteristics were explained by the “glaciation hypothesis.” In the hypothesis, the truffle populations in southern areas in Italy and Spanish acted as epibiotic species during glaciation and they spread northward along with their host trees as the ice receded (Bertault et al. 1998). The research results of García-Cunchillos et al. (2014) also supported this hypothesis. In this study, the Spanish populations of *T. melanosporum* were found to have higher genetic diversity than the Italian and French populations, with the species separated into two groups by the Iberian Mountain System (Table 1). Similarly, the Chinese *Tuber* species (*Tuber indium* complex) also showed a pattern consistent with a south to north migration in China (Wang et al. 2006a) (Table 1).

At the small geographic scale, host trees have been found to play a significant role in genotype distribution of *Tuber* truffles. *T. melanosporum* is a heterothallic species with two mating types. In some populations, only one mating type can be found under a single host tree (Murat et al. 2013) (Table 1). In other populations, Rubini et al. (2011a), Zampieri et al. (2012), and Linde and Selmes (2012) found biased distributions of two mating types of *Tuber* truffles under a single tree. Because sexual reproduction in *Tuber* species requires the mating between strains of two different mating types, the mycetophagous mammals play an important role for this to occur by bringing sexual partners into the proximity of root resident strains (Kataržytė and Kutorga 2011). Indeed, fine-scale genetic analyses have identified that multiple genotypes of *Tuber* truffles can exist under a single host tree (Murat et al. 2013). These results suggest that mycetophagous mammals are responsible for the observed long-distance dispersal, range expansion, gene flow, and genetic exchange between subpopulations of *Tuber* truffles.

The life cycles and productivity of *Tuber* truffles are impacted not only by their mycorrhizal host plants and mycetophagous mammals but also by the microbial community in truffle grounds. The microbial community structures have been found to differ between natural and cultivated truffle habitats, between productive and nonproductive plantations, and between different *Tuber* species and developmental stages of truffles. Belfiori et al. (2012) found that the diversity of ectomycorrhizal species is lower in cultivated plantations than in natural habitats, higher in *Tuber brumale*-colonized plants than that of *T. melanosporum*-colonized plants (Belfiori et al. 2012), and higher in productive plantations than in the nonproductive ones (De Miguel et al. 2014). In the *T. magnatum* natural habitats, the most abundant fungal species belong to Thelephoraceae, followed by Sebacinaceae, Inocybaceae, and Russulaceae (Murat et al. 2005; Leonardi et al. 2013). In fact, Thelephoraceae are frequently found in mature truffle orchards, but the *Hebeloma, Laccaria,* and *Russula* species seem to associate with unproductive grounds (De Miguel et al. 2014). Additionally, Barbieri et al. (2007) found that the total bacteria count associated with *T. magnatum* decreases during the maturation of ascocarps and that α-Proteobacteria is the major bacterial group associated with *T. magnatum* ascomata. Rivera et al. (2010) also investigated the total mesophilic microorganisms of *Tuber aestivum* and *T. melanosporum* ascocarps and found the presence of *Pseudomonas* genus, Enterobacteriaceae family, *Salmonella* spp., and *Listeria monocytogenes*. However, little information is currently available on the specific interaction of these microorganisms with truffles. This represents a fertile ground for future studies.

The studies of *Tuber* truffles provide a good model to study hypogeous macrofungi. The information of genetic structure and details of influence of associated mammals and microorganisms on truffles will help develop better conservation and cultivation of these macrofungi.

### 4.2. Fungi of Phallales

The Phallales is an order of Basidiomycota. Its members, the stinkhorn fungi, are well known for their morphologically unusual and brightly colored basidiomata as well as unpleasant odors associated with entomochory (Magnago et al. 2013). The order used to include two families Clathraceae and Phallaceae, with the shared and diagnostic feature of the peridium breaking down at maturity. Based on a phylogenetic analysis using molecular data, a study by Hosaka et al. (2006) suggested that the order Phallales should include six families (Clathraceae, Claustulaceae, Lysuriaceae, Phallaceae, Protophallaceae, and Trappeaceae) including some species with peridium not breaking down at maturity. Economically, the order Phallales includes many edible and medicinal species, such as the “veiled lady mushroom,” *Phallus indusiatus* (syn. *Dictyophora indusiata*). This cultivable mushroom is one of the most famous edible stinkhorn fungi with a high nutritive value and a delicious taste. Another mushroom of the genus, *Phallus rubicundus*, has been used as a traditional Chinese medicine. Ecologically, the stinkhorn fungi are mainly saprophytic fungi, play important roles as decomposers in forest ecosystem. Because of their saprophytic nature, these fungi are found in many areas in southern Asia, Africa, the Americas, and Australia, where they grow in woodlands and gardens in rich soil and well-rotted woody material.

The formation of fruiting bodies of stinkhorn fungi starts underground, and basidiospores are formed within a gleba. Then a receptacle, bearing the gleba, emerges from the peridium and extends above ground at maturity. As the gleba breaks down, the basidiospores are exposed in a gelatinous mass at the top of the stinkhorn (Glimn-Lacy and Kaufman 2006). Because the basidiospores cannot be dispersed in naturally due to the gelatinous matrix surrounding them, the insects play an important role in their dispersal. The insects are attracted to these fruiting bodies by the volatile substances they emit and forage the top of the fungi, with the spores released as excrement (Oliveira and Morato 2000). In this process, the insects consume the mucous matrix of the spores as nutrition while the spores remain intact and retain their germination ability in the digestive system until being excreted by the insects (Burr et al. 1996; Tuno 1998; Oliveira and Morato 2000). This pattern of spore dispersal is similar to the fly-pollinated angiosperm flowers (Johnson and Jürgens 2010). Consequently, the stinkhorn fungi are dispersed long distance through recruiting insects as spore dispersers with the insects benefiting from food of the mucilaginous glebae, creating opportunities for gene flow and gene exchange among populations of stinkhorn fungi. However, there is currently very limited data on the patterns of genetic variation and gene flow among geographic populations. Most current studies of stinkhorn fungi are on their artificial cultivation and analyses of their chemical and biological compounds.

### 4.3. Coprophilous macrofungi

Coprophilous fungi (dung fungi) are a special group of fungi that grow on animal feces, particularly those of herbivores. They break down dung for recycling nutrients and are dispersed by animals. Several hundred fungal species are known to grow on dung but relatively few of them form macroscopic fruiting bodies. These fungi develop and grow through a typical succession pattern that begins with phycomycetes, followed by ascomycetes (-cup and flask fungi), and basidiomycetes (Richardson 2002). Some of the ascomycete and basidiomycete dung fungi can fruit on dung, which are dispersed by animals. The cup and flask ascomycete spread spores in a typical pattern of most coprophilous fungi. The spores are produced in ascus and a high hydraulic pressure is built up, and upon reaching maturity, the spores are discharged, typically, toward light in the middle of the day, increasing their opportunity to reach new ecological niches (Richardson 2003). The spores of dung fungi need assistance of digestive juice of animals to break their dormancy in order to germinate on dung.

One of the best studied examples of dung macrofungi is the agaric genus *Coprinus* (basidiomycetes), the inky caps, which also include some saprophytic species on grassland and dead wood. These organisms have autodigestive chitinases and digest themselves (Nagy et al. 2013). Their basidiospores are black and released within a brown liquid to the dung during which the caps autolyze, typically within a few hours after basidiospores are formed (Kues 2000). Because of the mucous liquid around them, these spores cannot be dispersed by wind and may be scattered to a limited extent by rain. However, they can be disseminated by coprophilous insects, which forage on dung and carry basidiospores on their feet and bodies from one place to another (Brodie 1931). Because of the wide distributions of both dung materials and insects, the coprophilous fungi are also broadly distributed. Aside from their role as decomposers associated with dung wastes, the coprophilous fungi have little other known functions. However, several inky caps have been used as medicine for detumescence and to inhibit cancers. In addition, the coprophilous fungi also have been important materials for fungal research.

## 5. Conclusions and future directions

This paper reviewed the major types of macrofungi–animal interactions, with an emphasis on how the animals affected the reproduction and dispersal of representative macrofungi. A common feature of all these macrofungi is that they need animals to help produce and/or spread spores. However, the roles of these animals vary greatly, some only act as accidental transmission vectors while others are not only a disperser but also a required component of the fungal life cycle. The dispersal abilities and distribution ranges of the animals play a critical role in the fungal population genetic structures. Generally, the animals capable of long-distance dispersal typically show that their associated fungi have little or no geographic structure. In addition, we would like to point out other biotic factors, such as the microbial community of some macrofungi–animal interactions, likely play very important roles in mediating the interactions between animals and fungi and in the population dynamics of fungal communities.

The associations between macrofungi and plants are well known and widely acknowledged to have profound influences on the evolution and ecology of terrestrial life. Similarly, while not well acknowledged, the associations between macrofungi and animals also play important roles in natural ecosystem. In both the macrofungi–plant and macrofungi–animal interactions, many pivotal issues remain unknown. New research technology and methods may be useful to resolve them. First, the emerging methods in comparative omics (e.g., genomics and transcriptomics) and our increased understanding of the earth’s geological and climate histories may help us understand how the organisms have cooperated and coevolved. Second, the new methods of chemecology and landscape ecology can help us to understand how the relevant organisms attracted each other and interacted with each other to impact dispersal and distribution. Finally, in order to have a comprehensive understanding of the interactions between animals and macrofungi, more attention needs to be paid to all the organisms in the community, not just the macrofungi and/or the animals. In these niches, many bacteria, fungi, and plants and animals exist. However, we know very little about their diversity and roles. Understanding the species diversity, species differentiation, biogeography, and coevolution will greatly enhance our abilities to protect the inter-kingdom species interactions, increase the production of commercially and medically important macrofungi–animal interactions, and control agricultural and forestry pests.

## Disclosure statement

No potential conflict of interest was reported by the authors.
